# Wheat Ear Microbiome State as a Transferable Indicator of Within-Season Mycotoxin Risk

**DOI:** 10.1007/s00248-026-02837-9

**Published:** 2026-07-24

**Authors:** Oluwatobi Kolawole, Anne D. van Diepeningen, Josipa Grzetic Martens, Ane Arrizabalaga-Larrañaga, Lisa Black, Leo van Overbeek

**Affiliations:** 1 Department of Chemistry, University of Cambridge, Lensfield Rd, Cambridge, CB2 1EW UK; 2 Business Unit Biointeractions and Plant Health, Plant Sciences Group, Wageningen University & Research, Droevendaalsesteeg 1, Wageningen, 6708PB The Netherlands; 3 Wageningen Food Safety Research (WFSR), Wageningen University & Research, Akkermaalsbos 2, Wageningen, 6708 WB Netherlands; 4 Department of Applied Chemistry, University of the Basque Country (UPV/EHU), 20018, San Sebastian, Spain; 5 Agronomy and Plant Science Branch, Agri-Food and Biosciences Institute (AFBI), Belfast, Northern Ireland UK

**Keywords:** Ecological indicator, Wheat ear microbiome, Mycotoxin risk, Amplicon sequencing (16S/ITS), Decision support, Agroecosystem monitoring

## Abstract

Weather based models are central to mycotoxin risk assessment, but field level discrimination can be limited when environmental training data are sparse and models must transfer to locations not used during training. We tested whether wheat ear microbiome state provides a transferable biological indicator for within season classification of trichothecene contamination under strict spatial holdout, with relevance for food and feed safety. Across three winter wheat trial locations within a single production year, ears were sampled at BBCH 77 to 83, corresponding to mid to late grain filling, and paired bacterial 16 S rRNA gene and fungal ITS profiles were analysed alongside LC-MS/MS measurements of DON and T-2 + HT-2. Under leave one location out evaluation, microbiome informed models achieved substantially higher discrimination of higher versus lower contamination than station level weather summaries under identical spatial holdout. Weather features added little in the single season dataset, but simulation analyses indicated that environmental predictors become more learnable with temporal replication, supporting a future two stage framework in which early season weather context is refined by within season microbiome state. Portability screening and drop one ablation prioritised microbial indicators with sign consistent associations across locations and non-redundant predictive value. Together, these findings provide proof of concept that wheat ear microbiome state can support spatially transferable within season mycotoxin risk classification and targeted surveillance when environmental training data are limited.

**Supplementary Information:**

The online version contains supplementary material available at https://doi.org/10.1007/s00248-026-02837-9.

## Introduction

Mycotoxin contamination of cereal grain remains a persistent challenge for food and feed safety because toxin accumulation can vary substantially between fields, cultivars and production environments within the same growing season [1]. In wheat, trichothecene mycotoxins are of particular concern because they are chemically stable, can persist after harvest and may affect grain marketability even when disease symptoms are not severe [2]. Deoxynivalenol (DON) is among the most widely studied trichothecenes in wheat and is mainly associated with Fusarium head blight caused by species such as *Fusarium graminearum* and *F. culmorum* [1]. T-2 and HT-2 are also trichothecenes, but they are generally associated with different *Fusarium* species, including *F. langsethiae*, *F. sporotrichioides* and related members of the *Fusarium* complex [1, 2]. Together, DON and T-2 + HT-2 represent important safety indicators because their presence reflects both pathogen activity and the environmental conditions that favour toxin accumulation [3].

Current approaches for estimating mycotoxin risk rely largely on weather, crop development stage, agronomic information and known disease risk factors [4]. These approaches are valuable because moisture, temperature and humidity strongly influence *Fusarium* infection, fungal growth and toxin biosynthesis [2, 3]. Weather based models have therefore played an important role in FHB and mycotoxin decision support, particularly when they are calibrated with sufficient temporal replication, phenological timing and agronomic context [5]. However, weather-based predictors describe environmental suitability rather than the realised biological state of an individual field [6]. Their transferability can also be limited when training data are sparse, restricted to a single season or dominated by site level structure [7]. This distinction is important because two fields exposed to broadly similar weather may differ in pathogen pressure, cultivar response, fungicide history, local microclimate and resident microbial communities. As a result, field scale classification of mycotoxin risk can remain difficult, particularly when models are applied to locations that were not used during model development [8].

The wheat ear microbiome provides a potential biological layer for improving field scale risk assessment [9]. Bacterial and fungal communities on the ear occupy the same host tissue and microenvironment in which *Fusarium* infection and toxin accumulation occur [10]. These communities may therefore integrate local variation in host condition, pathogen pressure, microclimate and microbial interactions that is not fully captured by station level weather summaries [11]. The relationship between the wheat ear microbiome and mycotoxin risk is unlikely to be explained only by the abundance of a single toxin producing fungus [12]. Instead, toxin accumulation may reflect a broader community state involving *Fusarium*, competing fungi, opportunistic colonists, bacterial antagonists, host associated microbes and organisms that respond to changes in tissue condition during grain filling [13]. Previous work has shown that wheat associated microbial communities vary with plant development, management and disease status, and that naturally occurring bacteria and fungi can influence *Fusarium* growth, virulence and mycotoxin related outcomes [10–12].

The wheat ear microbiome may therefore provide information that is complementary to weather-based risk factors [14]. Rather than indicating only whether the environment was favourable for infection, microbiome profiles may capture the realised biological conditions present on the ear during grain filling [15]. For this concept to be useful in risk assessment, however, the signal must be portable across fields and locations [16]. Strong discrimination within a single location could reflect local structure, site specific taxa or overfitting rather than a transferable biological indicator [17]. A stricter test is therefore to develop the classification approach using some locations and evaluate it in a completely held out location [18]. Leave one location out (LOLO) validation provides such a test because it asks whether microbiome-based risk classification remains informative when applied to a field site that was not included during model development [19].

In this study, we evaluated whether wheat ear bacterial and fungal community profiles can serve as transferable indicators of within season trichothecene risk across three winter wheat trial sites on the island of Ireland. Wheat ears were sampled at BBCH 77 to 83, corresponding to mid to late grain filling, and paired bacterial 16 S rRNA gene and fungal ITS amplicon profiles were generated from the same plot level material used for LC-MS/MS quantification of DON and T-2 + HT-2 concentrations. The primary objective was to determine whether microbiome informed models improve spatially transferable classification of relative higher versus lower mycotoxin contamination compared with station level weather summaries under identical LOLO validation. A secondary objective was to identify genus level microbial indicators that showed directionally consistent associations with toxin concentrations across locations and to assess whether these indicators contributed unique predictive information using drop one ablation analysis. Because sampling was conducted at mid to late grain filling, the study was designed to assess within season risk classification rather than early season forecasting.

## Materials and Methods

### Field Design and Wheat Ear Sampling

Field experiments were conducted during a single production year in June 2024 at three winter wheat trial sites on the island of Ireland: Backweston, Crossnacreevy and Limavady. The sites formed part of a wider winter wheat performance trial in which cultivars were evaluated under fungicide treated and untreated management. For the present study, we focused on 11 cultivars that were common to all three locations, with both fungicide treated and untreated plots represented for each cultivar. No artificial *Fusarium* inoculation was applied, so the study evaluated naturally occurring variation in wheat ear microbiome composition and trichothecene contamination.

The final dataset comprised 262 plot level biological replicates, with 95 samples from Backweston, 75 from Crossnacreevy and 92 from Limavady. Here, n refers to the number of plot level composite samples included from each location. Each field plot measured 2 m × 10 m, corresponding to 20 m². Total plant number per plot was not counted, because plant density can vary with establishment, tillering and survival. Instead, sampling intensity was standardised by collecting 200 ears per sampled plot using a fixed within plot sampling scheme.

Wheat ears were sampled at BBCH 77 to 83, corresponding to mid to late grain filling. To capture within plot heterogeneity while reducing border effects, each plot was divided into five evenly spaced sampling sectors. Within each sector, 40 plants bearing ears were selected at random, giving 200 ears per plot. Ears from the five sectors were pooled to form one plot level composite sample.

Composite samples were transported to the laboratory on ice and stored at − 20 °C until processing. Ears were excised from stems, freeze dried to constant mass and milled to a fine homogeneous powder. Subsamples for mycotoxin analysis and DNA extraction were taken from the same milled material to ensure direct comparability between toxin measurements and microbiome profiles.

### Mycotoxin Quantification

DON, T-2 and HT-2 were quantified from freeze dried, milled wheat ear material using a validated LC-MS/MS multi mycotoxin method. Analyses were performed on a SCIEX QTRAP 6500 + triple quadrupole mass spectrometer coupled to a UHPLC system. For extraction, 5 g of homogenised material was weighed into an extraction vessel and extracted with 10 mL acetonitrile: acetic acid, 99:1, v/v. Samples were shaken for 1 min, magnesium sulphate was added and samples were shaken for a further 1 min. Extracts were cooled, centrifuged for 4 min at 4000 g, and 400 µL of supernatant was transferred to an LC vial containing 500 µL methanol and 20 µL isotope labelled internal standard mixture. The overall conversion factor from ng mL⁻¹ to µg kg⁻¹ was 4, accounting for extraction volume, subsampling and vial dilution.

Chromatographic separation and tandem mass spectrometry detection were performed in multiple reaction monitoring mode. Quantification used isotope dilution where labelled analogues were available and matrix matched external calibration for the remaining analytes. Analytes were considered identified when retention time agreement between sample and solvent standard was within 0.1 min, retention time agreement between native and labelled analogue was within 0.05 min, qualifier to quantifier ion ratio deviation was below 30% relative to the reference, and signal to noise ratio was greater than 3.

For individual toxin measurements, values below the limit of quantification but above the limit of detection were substituted with half the limit of quantification, whereas values below the limit of detection were set to zero. The combined endpoint T-2 + HT-2 was calculated as the sum of processed individual T-2 and HT-2 concentrations for each sample.

Each analytical batch included extraction blanks, solvent blanks and fortified quality control materials. Limits of quantification were 5 µg kg⁻¹ for DON, 2.5 µg kg⁻¹ for T-2 and 5 µg kg⁻¹ for HT-2. Method recovery was 76% for DON, 81% for T-2 and 87% for HT-2. Within laboratory reproducibility was 11% for DON, 17% for T-2 and 16% for HT-2. Expanded measurement uncertainty was 22% for DON, 34% for T-2 and 31% for HT-2. Matrix effects were − 40% for DON, − 30% for T-2 and − 10% for HT-2. Location level summaries of toxin concentrations are reported in the Results.

### DNA Extraction, Amplicon Generation and Sequencing

Total genomic DNA was extracted from freeze dried, homogenised wheat ear material using the MolGen PurePlant DNA extraction kit according to the manufacturer’s protocol for plant tissues. DNA concentration and purity were assessed spectrophotometrically, and DNA integrity was verified using 1% agarose gel electrophoresis. Purified DNA was shipped to BMKGENE, Germany, for PCR amplification, amplicon library preparation and sequencing.

Bacterial communities were profiled using the V3 to V4 region of the 16 S rRNA gene with primers 341 F and 805R. Fungal communities were profiled using the ITS1 region with primers ITS1F and ITS2. For 16 S amplification, peptide nucleic acid clamps targeting chloroplast and mitochondrial 16 S rRNA genes were included to reduce amplification of host organelle DNA. Primer sequences, PCR conditions and PNA clamp details are provided in Supplementary Table S1.

For each marker set, five extraction blanks and five PCR no template controls were included and carried through amplification, sequencing and downstream processing. A fungal mock community was also sequenced as a positive control for ITS performance and taxonomic assignment. Libraries were sequenced on an Illumina MiSeq platform using 2 × 300 bp paired end chemistry. Sequencing depth, read retention, ASV counts and final genus level feature counts are summarised in Supplementary Table S2.

### Amplicon Sequence Processing and Contaminant Filtering

Demultiplexed 16 S and ITS reads were processed in R version 4.3.3 using DADA2 version 1.30.0 to infer amplicon sequence variants, hereafter ASVs. Forward and reverse reads were quality filtered and trimmed before dereplication, error rate learning, ASV inference, paired read merging and chimera removal. Field samples and negative controls were processed using the same workflow so that contaminant assessment was performed in the same ASV feature space. Full filtering parameters are provided in Supplementary Table S2.

Taxonomy was assigned using SILVA SSU v138.2 for 16 S and the UNITE fungal ITS reference database for ITS. Chloroplast and mitochondrial assignments were removed from the 16 S dataset, and non-fungal assignments were removed from the ITS dataset before downstream analysis. Putative reagent contaminants were identified using the prevalence method in the decontam R package by comparing detection frequency in negative controls with detection frequency in true samples. ASVs flagged as contaminants were removed before genus aggregation, centred log ratio transformation and model training.

Samples were randomised across extraction and sequencing runs for both marker sets. Batch and run structure were examined to ensure that they did not align with location. Additional checks comparing community structure and the main modelling feature axes across sequencing runs did not indicate that batch or sequencing run explained the discrimination patterns observed under spatial validation.

### Weather Predictors

Hourly weather data were obtained from site level meteorological stations. Weather predictors were summarised over fixed windows of 7, 14, 21 and 30 days before sampling. These summaries included cumulative rainfall, temperature summaries, hours with relative humidity of at least 90%, and growing degree days. Weather features were centred and scaled using the training data within each validation fold and then applied unchanged to the held-out location.

The modelling unit was the plot level sample. Each plot record therefore had both microbiome predictors and matched meteorological predictors. However, microbiome profiles were measured directly from plot level composite samples, whereas meteorological predictors were derived from site level weather records and assigned to plot samples according to location and sampling window.

To quantify the degree to which weather summaries were structured by location, intraclass correlation coefficients, hereafter ICCs, were estimated for weather variables. Higher ICC values indicate stronger location level structuring relative to within location variation.

### Microbiome Feature Construction

Cleaned ASV tables were aggregated to genus level separately for bacterial and fungal datasets. In this study, abundance refers to sequence count abundance after filtering, taxonomic assignment and genus level aggregation. Genus level count tables were transformed using a centred log ratio, hereafter CLR, transformation with a pseudocount of 1 to account for the compositional nature of amplicon sequencing data.

For predictive modelling, principal component analysis was applied separately to the CLR transformed bacterial and fungal genus matrices. The first 15 principal components from each marker set were retained and concatenated to form the microbiome feature block. All preprocessing steps that learned parameters, including CLR centring, scaling and principal component loadings, were estimated using training locations only and then applied unchanged to the held-out location.

### Prediction Task and Model Comparison

The primary prediction task was binary classification of relative higher versus lower mycotoxin contamination for DON and T-2 + HT-2. In each leave one location out, hereafter LOLO, validation split, the classification threshold was defined as the median toxin concentration in the training locations only. This threshold was then applied unchanged to the held-out location. This approach avoided using information from the test location when defining the response variable and produced a balanced proof of concept classification task. The resulting labels represent relative mycotoxin risk within the study dataset rather than exceedance of a fixed regulatory threshold.

LOLO validation was used to test spatial transferability. In each fold, models were trained on two locations and evaluated on the third, completely held out location. This design provides a stricter test than random cross validation because it asks whether the classifier remains informative when applied to a site that was not used during model development.

Three predictor configurations were evaluated for each toxin endpoint: weather only, microbiome only, and combined weather plus microbiome. The combined model used direct concatenation of weather and microbiome features without interaction terms. Models were fitted using penalised logistic regression with elastic net regularisation. This approach was selected because it is suited to correlated, high dimensional predictors and moderate sample sizes, while remaining more constrained than highly flexible machine learning models. The regularisation parameter was selected using internal cross validation within the training locations. Performance was summarised using the area under the receiver operating characteristic curve, hereafter AUC. AUC was used because it measures how well the model ranks higher contamination samples above lower contamination samples across classification thresholds.

### Threshold and Covariate Sensitivity Analyses

Sensitivity analyses were performed to assess whether the main conclusion depended on the median based class definition. The LOLO analysis was repeated using alternative training fold thresholds, including the 75th percentile and the upper tertile. These thresholds were also calculated from training locations only and then applied unchanged to the held-out location.

As an additional robustness check, cultivar identity and fungicide treatment were included as covariates in the modelling framework. Their inclusion did not materially change discrimination performance, with absolute AUC changes of approximately 0.01 across toxin endpoints, locations and model configurations. These covariates were therefore excluded from the primary models for parsimony.

### Portable Genus Screen and Drop One Ablation

To identify microbial signals that were stable across spatial contexts, associations between CLR transformed genus abundances and toxin concentrations were quantified within each location using Spearman rank correlation. Genera were retained as portable candidates when they met three criteria: prevalence of at least 70% within each location, absolute Spearman correlation of at least 0.30 within each location, and directionally consistent associations across all three locations with no sign reversals. A stricter screen requiring 100% prevalence within each location yielded a highly similar set of genera, indicating limited sensitivity to the prevalence threshold.

Drop one ablation analysis was then used to distinguish stable association from unique predictive contribution. For each candidate genus, the genus was removed from the dataset, and the full modelling workflow was recomputed. This included recomputing the CLR transformation, refitting principal component analysis using training samples only, projecting held out samples into the refitted component space, retraining the elastic net classifier and recomputing held out AUC. Ablation effects were summarised as ΔAUC = AUC of the full model minus AUC after genus removal. Positive ΔAUC values indicate that model discrimination decreased after removing the genus, consistent with unique predictive contribution. Near zero or negative values indicate redundancy or limited added value in the presence of other microbiome features.

### Uncertainty Estimation and Permutation Testing

Uncertainty in held out AUC was estimated using nonparametric bootstrap resampling of the held-out test set. For each toxin endpoint, predictor configuration and held out location, paired class labels and predicted probabilities were resampled with replacement 2000 times. The 95% confidence interval was defined using the 2.5th and 97.5th percentiles of the bootstrap AUC distribution. Bootstrap replicates containing only one class were excluded from percentile estimation.

Permutation testing was used to assess whether observed discrimination exceeded chance expectations. For each held out location and predictor configuration, class labels were randomly permuted within the training set only. The model was then refitted using the permuted labels, and predicted probabilities were generated for the unchanged held out location. This procedure was repeated 1000 times. One-sided permutation p values were calculated as the proportion of null AUC values greater than or equal to the observed AUC, with a plus one correction. These p values test performance against a random label null and were not interpreted as direct statistical comparisons between microbiome and weather models. Bootstrap 95% confidence intervals and permutation p values for held out AUC are reported in the Supplementary Data.

### Simulation based Design Analysis

A simulation-based design analysis was used to examine when environmental predictors might become informative under spatial transfer and how temporal replication could affect integrated weather microbiome prediction. The analysis evaluated a two-stage framework in which an early season weather component was refined by later microbiome information. Scenarios varied the number of years, locations and samples per location per year, and performance was summarised using spatial holdout AUC. Full simulation assumptions, parameter settings and scenario definitions are provided in the Supplementary Methods.

## Results

### Mycotoxin Concentrations Varied Across Locations and Plots

DON and T-2 + HT-2 were detected at variable concentrations across the three trial locations, indicating naturally occurring differences in trichothecene contamination within the study year. No artificial *Fusarium* inoculation was applied, so the observed toxin profiles reflect natural field variation under the trial conditions. Concentrations were not uniformly low, which is expected because the dataset included multiple cultivars with differing susceptibility as well as both fungicide treated and untreated plots. Concentration distributions differed among locations and also showed within location variation among plot level composite samples.

To provide concentration context for the classification analyses, toxin summaries are reported by location in Table 1. The combined T-2 + HT-2 endpoint was calculated as the sum of processed individual T-2 and HT-2 concentrations for each sample, as described in the Methods. These concentration values were used to define higher versus lower contamination classes within each leave one location out training fold. DON reached 3000 µg kg⁻¹ and T-2 + HT-2 reached 480 µg kg⁻¹ in naturally contaminated plot level samples.

**Table 1:** Mycotoxin concentrations in plot level wheat ear samples by location

Toxin	Location	*n*	Median, µg kg⁻¹	IQR, µg kg⁻¹	Min–max, µg kg⁻¹
DON	Backweston	95	120	76–283	25–3000
DON	Crossnacreevy	75	207.5	180–290	120–600
DON	Limavady	92	80.5	49.5–143	18–433
T-2 + HT-2	Backweston	95	50	36–89	3–480
T-2 + HT-2	Crossnacreevy	75	20.5	13.8–37.8	10–65
T-2 + HT-2	Limavady	92	31.5	16.2–50.2	3–198

Values are reported as µg kg⁻¹. IQR refers to the interquartile range, and min–max refers to the minimum and maximum observed concentrations. T-2 + HT-2 was calculated as the sum of processed individual T-2 and HT-2 concentrations for each sample. Individual toxin values below analytical thresholds were handled as described in the Methods. n refers to the number of plot level composite samples included from each location.

### Microbiome Profiles Improved Transferable Classification of Mycotoxin Risk

**Fig. 1:**
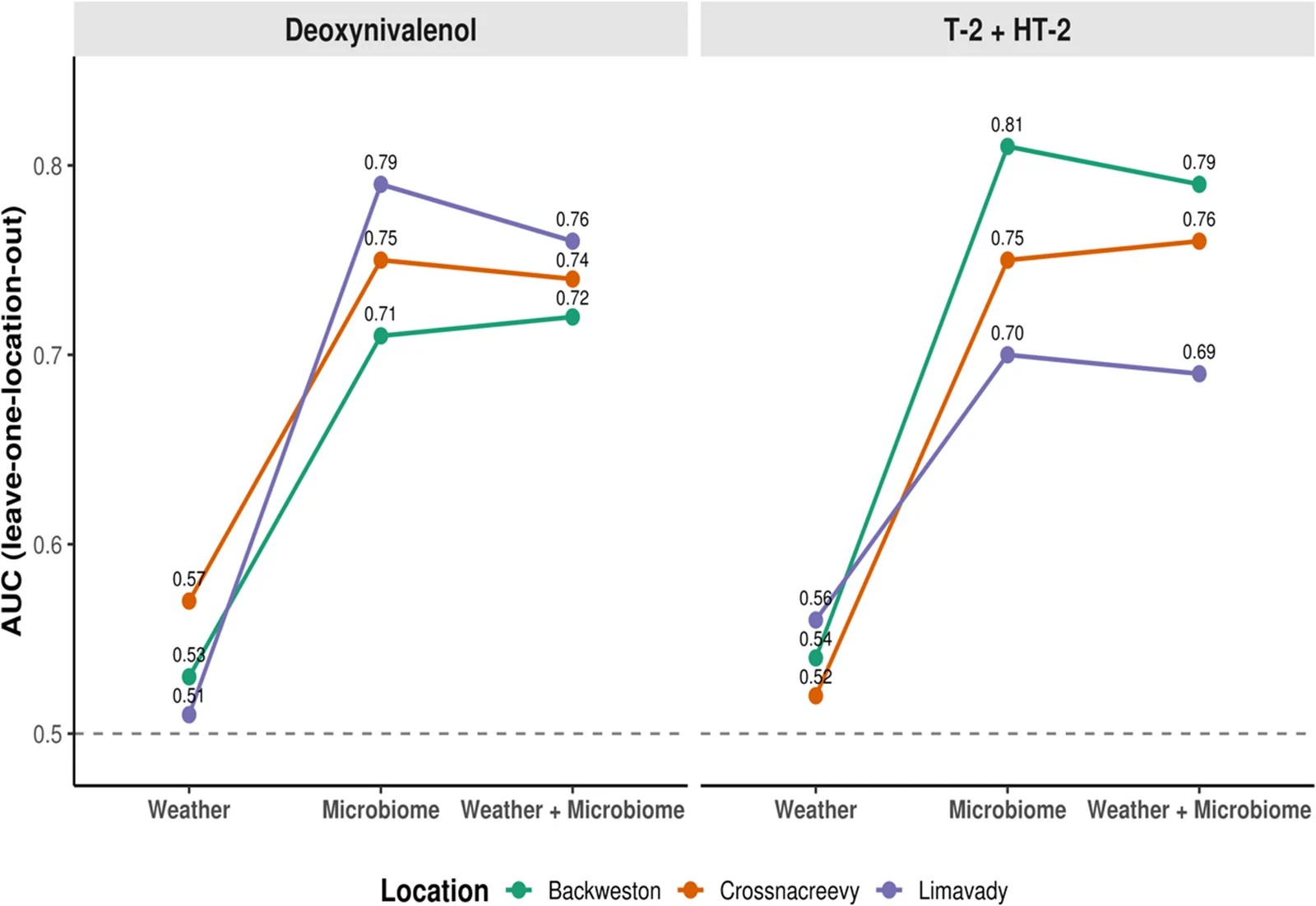
Transferable classification of trichothecene contamination under spatial holdout. Held out AUC for weather only, microbiome only and combined models under leave one location out validation for DON and T-2 + HT-2. The dashed line indicates chance discrimination, AUC = 0.5. Bootstrap 95% confidence intervals are reported in the Supplementary Data

Under strict LOLO validation, microbiome informed models consistently outperformed weather only models for both toxin endpoints (Fig. 1). For DON, weather only models showed limited discrimination, with held out AUC values of 0.53 in Backweston, 0.57 in Crossnacreevy and 0.51 in Limavady. In contrast, microbiome only models achieved higher held out discrimination, with AUC values of 0.71, 0.75 and 0.79 across the same locations. Combined weather plus microbiome models performed similarly to microbiome only models, with AUC values of 0.72, 0.74 and 0.76.

A similar pattern was observed for T-2 + HT-2 (Fig. 1). Weather only models again showed weak discrimination, with AUC values of 0.54, 0.52 and 0.56 across Backweston, Crossnacreevy and Limavady. Microbiome only models achieved higher AUC values of 0.81, 0.75 and 0.70, while combined weather plus microbiome models achieved AUC values of 0.79, 0.76 and 0.69. Across both toxin endpoints, wheat ear microbiome profiles therefore provided a stronger transferable signal than station level weather summaries when models were trained on two locations and tested on the third. The combined models did not provide consistent improvement over microbiome only models, indicating that station level weather summaries added limited additional discriminatory information once microbiome community state had been included.

### Model Performance was Robust to Threshold Definition and Covariate Adjustment

The primary classification task used the training fold median toxin concentration to define higher versus lower contamination. This threshold was chosen using training locations only and was then applied unchanged to the held-out location, avoiding information leakage from the test location. To assess whether the main conclusion depended on this median based definition, the leave one location out analysis was repeated using alternative thresholds, including the training fold 75th percentile and the upper tertile. Individual toxin endpoints were also evaluated where relevant. Across these sensitivity analyses, microbiome informed models remained superior to weather only models, indicating that the main conclusion was not driven by the median split. Including cultivar identity and fungicide treatment as additional covariates also did not materially change model performance. Across toxin endpoints, held out locations and model configurations, absolute changes in AUC were approximately 0.01. The relative ranking of model classes remained unchanged, with microbiome informed models outperforming weather only models and combined models showing no consistent gain over microbiome only models. These results indicate that the microbiome signal was not simply explained by cultivar or fungicide treatment structure.

### Seasonal Weather Summaries were Strongly Site Linked and Added Little under Spatial Transfer

**Fig. 2:**
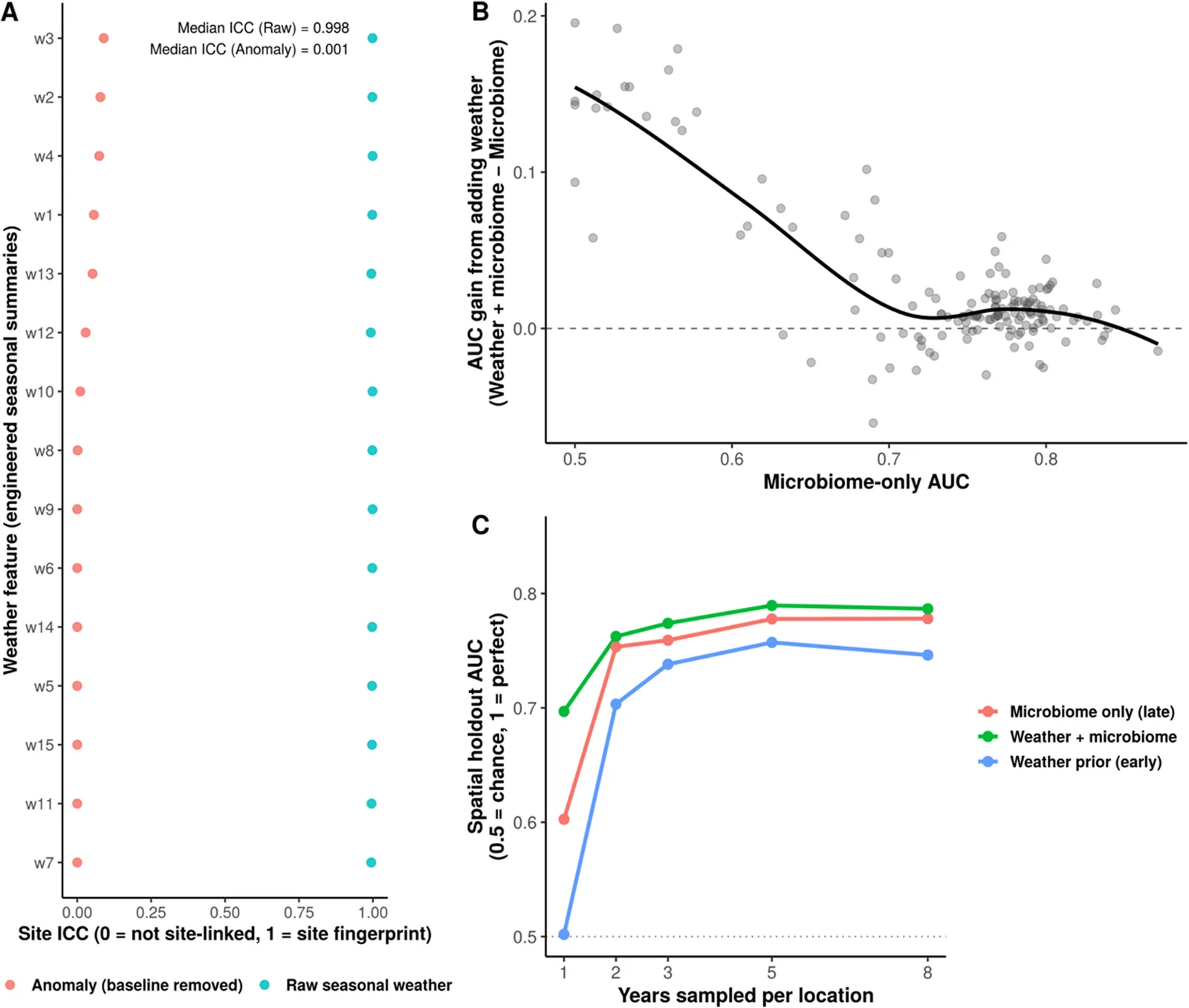
Weather structure and simulation-based design analysis. **A**, Intraclass correlation coefficients, ICCs, for raw and anomaly transformed weather features. **B**, AUC gain from adding weather to microbiome models, calculated as combined model AUC minus microbiome only AUC. The dashed line indicates no gain. **C**, Simulation analysis showing how spatial holdout AUC changes as temporal replication increases in a two-stage weather microbiome framework

Although hybrid models combining microbiome and weather predictors are conceptually attractive, weather summaries showed limited discrimination under strict spatial holdout and did not consistently improve performance beyond microbiome only models (Fig. 1). To understand this pattern, the structure of the engineered weather features was examined across locations (Fig. 2A). Raw seasonal weather summaries showed very high site level intraclass correlation coefficients (ICC), with a median ICC of 0.998. This indicates that these variables behaved primarily as location level descriptors rather than plot level predictors. When weather features were expressed as anomalies relative to location means, the site fingerprint was effectively removed, with the median ICC decreasing to 0.001 (Fig. 2A). However, this transformation did not restore predictive discrimination. This suggests that the low AUC of weather only models was not due only to site identity but also reflected limited plot level discriminatory information in the station-based weather summaries.

The marginal benefit of adding weather also depended on the strength of the microbiome signal (Fig. 2B). Across held out evaluations, the gain in AUC from adding weather to the microbiome model was generally small and tended to diminish as microbiome only performance increased. Thus, within this single season dataset, weather information contributed limited additional predictive value once wheat ear microbiome state had been measured.

Because the empirical dataset covered only one production year, simulation-based analyses were used to examine when environmental predictors might become informative under spatial transfer. Simulations parameterised from the observed data showed that when training data were limited to a single year, transferable environmental signal remained weak and final discrimination was driven mainly by the microbiome component, consistent with the empirical LOLO results (Fig. 2C). As additional years were incorporated, the contribution of early season weather became more learnable under spatial holdout, with clearer gains emerging once training covered approximately three years. Performance then approached saturation at approximately 40 to 50 samples per location per year across eight locations over three years (Fig. 2C; Supplementary Figure S3). Together, these findings indicate that weather and microbiome predictors captured different aspects of mycotoxin risk: weather provided broad environmental context, whereas within season microbiome profiles captured field level biological state at sampling.

### Portable Microbial Indicators Showed Consistent Associations Across Locations

**Fig. 3:**
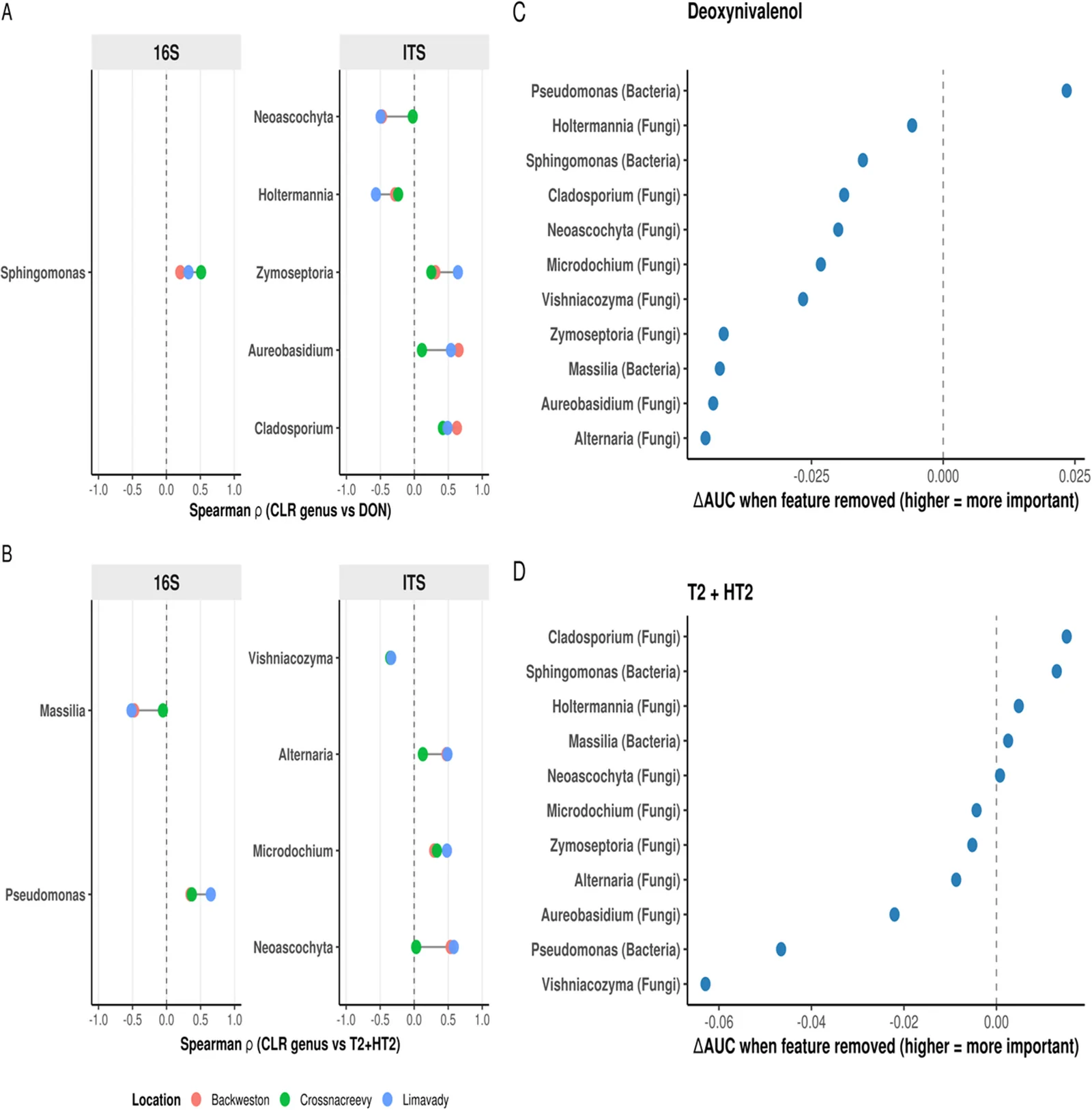
Portable genus level indicators and predictive contribution.** A**,** B**, Within location Spearman correlations between CLR transformed genus abundance and toxin concentration for genera showing directionally consistent associations across locations. **C**,** D**, Drop one ablation showing the change in held out AUC after removing each candidate genus. Positive ΔAUC indicates loss of discrimination after genus removal; values near zero or below zero indicate redundancy or limited unique contribution

The microbiome signal was next examined to determine whether it reflected broad community structure or a smaller set of microbial indicators that were stable across locations (Fig. 3). Genus level associations between CLR transformed abundance and toxin concentration were calculated separately within each location. Genera were retained as portable candidates when they were prevalent within each location, showed at least moderate association strength and had directionally consistent associations across all three locations. *Fusarium* was detected in the sequencing dataset, but it did not meet these combined portability criteria as strongly as the genera shown in Fig. 3.

For DON, the portable candidate set included fungal genera such as *Cladosporium*, *Aureobasidium* and *Zymoseptoria*, alongside bacterial indicators including *Sphingomonas* (Fig. 3A). For T-2 + HT-2, the portable set included bacterial genera such as *Pseudomonas* and *Massilia*, together with fungal genera including *Alternaria* and *Microdochium* (Fig. 3B). These patterns indicate that the transferable signal was not restricted to a single fungal taxon but instead reflected a broader bacterial and fungal community state associated with toxin contamination.

### Drop One Ablation Identified Taxa with Unique Predictive Contribution

Directionally consistent association does not necessarily imply unique predictive value, because correlated taxa may carry redundant information. Drop one ablation was therefore used to test whether removing individual candidate genera reduced held out discrimination under the full LOLO modelling pipeline (Fig. 3C, D).

For DON, removal of *Pseudomonas* produced the largest reduction in AUC, with smaller positive contributions from *Holtermannia* and *Sphingomonas* (Fig. 3C). This contribution was endpoint specific, because *Pseudomonas* did not show the same unique contribution to T-2 + HT-2 classification. Several other candidate genera showed near zero or negative ΔAUC, suggesting redundancy or limited additional value in the presence of stronger predictors. For T-2 + HT-2, positive ablation effects were strongest for *Cladosporium* and *Sphingomonas*, with smaller positive contributions from *Holtermannia* and *Massilia* (Fig. 3D). Again, several genera showed little or no unique contribution after accounting for the broader microbiome feature set.

Together, the portability screen and ablation analysis show that stable association and predictive contribution were related but not identical properties (Fig. 3). Some genera showed consistent associations with toxin concentrations across locations, whereas others contributed more strongly to spatially transferable classification once the full community model was considered.

## Discussion

This study provides proof of concept that wheat ear microbiome state can serve as a transferable biological indicator of within season trichothecene risk. The central finding is that paired bacterial and fungal community profiles supported spatially transferable classification of DON and T-2 + HT-2 risk under strict LOLO validation, whereas station level weather summaries alone were less informative in this single season setting. This does not diminish the value of weather-based risk modelling. Rather, it shows that microbiome data can provide a complementary biological layer that captures realised field conditions not fully represented by seasonal weather summaries [20].

Weather based forecasting has been central to FHB and mycotoxin risk assessment for several decades, but its performance depends strongly on the scale, timing and diversity of the data used for model development [21]. Foundational FHB models were built from multi location year datasets and identified weather windows around anthesis, particularly rainfall, humidity and temperature, as informative predictors of epidemic risk [1]. DON prediction frameworks have also shown value when weather is combined with agronomic information such as cultivar susceptibility, previous crop, tillage and phenology [2, 3]. However, these studies also show why weather-based prediction can be difficult to transfer [22]. Empirical models are often highly dependent on the environments, years and management systems represented in the training data, and their use in new regions or production contexts requires careful validation [2, 4]. Recent work has similarly shown that even large weather and agronomic datasets can perform well in internal validation while struggling to predict years excluded from model training [4]. Thus, weather variables are biologically relevant and operationally useful, but they may provide limited transferable discrimination when training data are sparse, temporally narrow or dominated by site level structure [23].

The present weather results fit this broader literature. Raw seasonal weather summaries were strongly site linked, and anomaly transformation removed this location fingerprint but did not restore discrimination (Fig. 2A). This suggests that the weak performance of weather only models was not simply due to confounding by location. Rather, one production year across three locations provided limited learnable environmental variation for spatial transfer [24]. This interpretation is consistent with recent modelling work showing that DON prediction can be improved by adding agronomic and site-specific information to weather, but that predicting years not represented in the training data remains difficult [4]. In our setting, weather described broad environmental conditions favourable for DON and T-2 + HT-2 risk, whereas the wheat ear microbiome provided a plot level biological readout of the realised state at sampling. Because weather summaries were derived from site level records and assigned to plot level samples, they could not fully resolve plot to plot variation in cultivar response, fungicide treatment, pathogen pressure, canopy microclimate or resident microbial community state. These sources of local biological variation may be captured more directly by the microbiome.

The simulation analysis gives this finding a practical forward path. It indicates that weather information becomes more learnable under spatial holdout as temporal replication increases, with clearer gains once training spans multiple years (Fig. 2B, C) [25]. This supports a two-stage framework for future decision support: early season weather and agronomic data provide a prior estimate of environmental risk, while later microbiome measurements refine that estimate using field level biological state. This suggests that future studies should prioritise multiyear, multi-location datasets with standardised microbiome sampling, toxin measurements, crop stage information and weather summaries, rather than relying only on adding more variables to a single season dataset [26]. The simulation results suggest that approximately three years of data, with around 40 to 50 samples per location per year across multiple locations, would provide a stronger basis for testing integrated weather microbiome models [27].

The timing of sampling defines the current scope of application. Ears were sampled at BBCH 77 to 83, so the present study supports within season risk classification at mid to late grain filling rather than early season forecasting. This distinction is important for implementation. The approach should not yet be viewed as a direct fungicide decision tool, because fungicide applications for FHB are most time sensitive around heading and flowering [28]. Its more immediate value is likely to be in targeted surveillance, prioritising plots or fields for LC MS/MS testing, harvest risk stratification, grain segregation and post-harvest destination decisions [29]. Future work should test additional key developmental stages to determine when the microbiome signal first becomes detectable and when it is most useful for decision support [30].

The microbial indicators identified here are best interpreted as signatures of community state rather than as isolated causal agents. DON and T-2 + HT-2 accumulation are shaped by toxin producing *Fusarium* species, host condition, infection timing, local microclimate and interactions with other fungi and bacteria [31]. A microbiome perspective on FHB is therefore appropriate because *Fusarium* interacts with the cereal microbiome across residues, roots, leaves, heads and grain, and because several bacterial and fungal taxa can influence pathogen growth, disease development or mycotoxin related outcomes [5]. The portable taxa identified in this study, including genera such as *Cladosporium*, *Aureobasidium*, *Zymoseptoria*, *Sphingomonas*, *Pseudomonas*, *Massilia*, *Alternaria* and *Microdochium*, should therefore be viewed as indicators of ecological conditions associated with toxin risk (Fig. 3) [32].

*Fusarium* was detected in the sequencing dataset, but it did not emerge among the strongest portable genus level indicators under the prevalence, association strength and directional consistency criteria used here. This does not imply that *Fusarium* was unimportant. DON, T-2 and HT-2 remain *Fusarium* associated toxins, and *Fusarium* biology is central to their interpretation [33]. Amplicon sequencing, however, measures relative marker abundance in a mixed community and is not equivalent to culture-based isolation, targeted pathogen quantification or direct measurement of toxin biosynthesis [34]. Toxin accumulation may depend on species or strain identity, infection timing, fungal activity and host tissue conditions rather than relative sequence abundance alone [35]. It is therefore plausible that non-Fusarium taxa provide stronger indicator information if they track the ecological state in which *Fusarium* infection and toxin production occur [36].

This interpretation also helps explain why different taxa contributed to DON and T-2 + HT-2 classification. The two endpoints may reflect different *Fusarium* species, infection windows, environmental sensitivities and microbial interactions [37]. For example, phyllosphere fungi such as *Cladosporium*, *Alternaria* and *Aureobasidium* commonly occupy cereal head and grain niches, while bacterial genera such as *Pseudomonas* and *Sphingomonas* may reflect antagonistic, competitive or host associated processes [38]. Their predictive value does not require them to be toxin producers [39]. Instead, they may mark biological states that are more or less favourable to toxin accumulation [40]. These taxa should therefore be treated as candidates for validation in controlled experiments, longitudinal field sampling and targeted molecular assays [41].

A practical next step is to move from broad amplicon sequencing to reduced indicator panels. Amplicon profiling is well suited for proof-of-concept discovery because it surveys the wider bacterial and fungal community without prior assumptions [42]. Operational decision support, however, will require faster, cheaper and more standardised workflows [43]. A further practical consideration is turnaround time, because implementation would depend on whether sampling, DNA extraction, assay processing and reporting can be completed within the relevant pre harvest decision window. The portable indicators identified here provide a starting point for qPCR, multiplex or other targeted assays that could be benchmarked against full community sequencing and LC MS/MS toxin measurements [44]. Such assays would also make it easier to test sampling density, turnaround time and field scale deployment [45].

Several limitations define the scope of the present study. The dataset covers one production year, so transferability was tested spatially rather than temporally. Multiyear validation is needed before the approach can be generalised across contrasting seasonal conditions [46]. Larger datasets with broader toxin distributions will also be needed to train and validate models based on fixed concentration thresholds, regulatory exceedance or quantitative prediction of toxin concentrations. The sampling stage means that the current signal is most relevant for within season classification and surveillance rather than early intervention [47]. Finally, each sample was a composite of 200 ears from a plot, which is appropriate for proof-of-concept testing but does not yet define the number of samples required per hectare in commercial fields [48]. Future studies should standardise sampling patterns, compare plot and field scale composites, include additional developmental stages and evaluate performance across cultivars, management systems and disease pressure gradients [49].

## Conclusion

This study provides proof of concept that paired wheat ear bacterial and fungal microbiome profiles can support spatially transferable, within season classification of trichothecene risk under LOLO validation. The approach is not intended to replace weather-based risk models, but to add a realised biological layer to existing environmental risk assessment. Future work should test this framework across multiple years, locations and developmental stages, with the aim of integrating early season weather and agronomic context within season microbiome indicators for field level mycotoxin surveillance and decision support.

## Supplementary Information

Below is the link to the electronic supplementary material.

Supplementary File 1 (CSV 2.34 KB)

Supplementary File 2 (DOCX 93.5 KB)

## Data Availability

Raw 16 S and ITS amplicon reads have been deposited in the Sequence Read Archive at NCBI under BioProject PRJNA1419250. Processed data products and derived analysis inputs are archived at https://doi.org/10.5281/zenodo.18622238**.** All analysis scripts required to reproduce the results and figures are available at https://doi.org/10.5281/zenodo.1862223. All analyses were conducted in R (v4.3.3) using DADA2 (v1.30.0) and glmnet (v4.1-8).
