# Supplementary material for: Wheat Ear Microbiome State as a Transferable Indicator of Within-Season Mycotoxin Risk
Source: Microb Ecol. 2026 Jul 24;89:194. doi: 10.1007/s00248-026-02837-9 (PMC13710519; doi:10.1007/s00248-026-02837-9)
Supplement: Supplementary file 1 [file 248_2026_2837_MOESM2_ESM.docx]

**Wheat ear microbiome state as a transferable indicator of within-season mycotoxin risk**

**Oluwatobi Kolawole^1,2 ☑^, Anne D. van Diepeningen^2^, Josipa Grzetic Martens^3^, Ane Arrizabalaga Larranaga^3^, Lisa Black^4^, Leo van Overbeek^2^**

^1^Department of Biochemistry, Sanger Building, University of Cambridge, Sanger Building, 80 Tennis Ct Rd, Cambridge CB2 1GA, United Kingdom

^2^Business Unit Biointeractions and Plant Health, Plant Sciences Group, Wageningen University & Research, Droevendaalsesteeg 1, 6708PB Wageningen, The Netherlands.

^3^Wageningen Food Safety Research (WFSR), Wageningen University & Research, Akkermaalsbos 2, 6708 WB Wageningen, Netherlands

^4^Agronomy and Plant Science, Agri-Food and Biosciences Institute, Belfast, Northern Ireland, United Kingdom.

**☑** corresponding author: tk698@cam.ac.uk

**Supplementary Table S1. Primer sequences, PCR conditions and PNA clamp details used for 16S rRNA gene and ITS amplicon sequencing.**

This table reports the marker region, primer names, primer sequences, amplification conditions and peptide nucleic acid clamp information used for bacterial and fungal community profiling.

| **Marker** | **Target region** | **Primer** | **Sequence 5′–3′** | **Notes** |
| --- | --- | --- | --- | --- |
| 16S | V3–V4 | 341F | CCTACGGGNGGCWGCAG | Bacterial community profiling |
| 16S | V3–V4 | 805R | GACTACHVGGGTATCTAATCC | Bacterial community profiling |
| ITS | ITS1 | ITS1F | CTTGGTCATTTAGAGGAAGTAA | Fungal community profiling |
| ITS | ITS1 | ITS2 | GCTGCGTTCTTCATCGATGC | Fungal community profiling |
| 16S | Chloroplast PNA clamp | pPNA | GGCTCAACCCTGGACAG | Host organelle suppression |
| 16S | Mitochondrial PNA clamp | mPNA | GGCAAGTGTTCTTCGGA | Host organelle suppression |

**Supplementary Table S2. Sequencing depth, read retention and feature summary for 16S and ITS datasets.**

Depth values refer to per sample non chimeric merged reads after DADA2 processing. The table also reports denoised reads, merged reads, read retention, ASV counts and assigned genus counts used for downstream analyses.

| **Metric** | **16S** | **ITS** |
| --- | --- | --- |
| Samples | 262 | 262 |
| Denoised forward reads | 16,060,714 | 13,110,742 |
| Denoised reverse reads | 16,003,127 | 13,110,061 |
| Merged reads | 14,548,430 | 12,904,231 |
| Non-chimeric reads | 14,548,430 | 12,904,231 |
| Merge retention vs forward, % | 90.6 | 98.4 |
| Merge retention vs reverse, % | 90.9 | 98.4 |
| Non-chimeric retention vs merged, % | 100.0 | 100.0 |
| Raw ASVs | 256,749 | 9,863 |
| Non-chimeric ASVs | 256,749 | 9,863 |
| Assigned genera | 1,205 | 370 |
| Minimum depth | 73,832 | 81,890 |
| Q1 depth | 95,657 | 106,496 |
| Median depth | 111,937 | 120,866 |
| Mean depth | 138,557 | 122,897 |
| Q3 depth | 128,499 | 134,980 |
| Maximum depth | 598,162 | 219,581 |

**Table note:** Depth values refer to per-sample non-chimeric merged reads after DADA2 processing. ASVs were taxonomically assigned and aggregated to genus level for downstream analyses.

# Supplementary Methods: Simulation Analyses

## Weather site-identifiability analysis

Before evaluating predictive performance, we assessed whether seasonal weather summaries behaved as transferable risk covariates or primarily as location identifiers. For each weather feature, site-level structure was quantified using a one-way random-effects model:

$$w_{ij}=\mu+u_{i}+\epsilon_{ij}$$

where $w_{ij}$ is the value of a weather feature for sample $j$ at location $i$, $\mu$ is the overall mean, $u_{i}$ is the location effect, and $\epsilon_{ij}$ is residual variation. The random components were defined as

$$u_{i}\sim N\left( 0,\sigma_{\mathrm{site}}^{2} \right), \epsilon_{ij}\sim N\left( 0,\sigma_{\mathrm{res}}^{2} \right).$$

The site intraclass correlation coefficient was calculated as

$$\mathrm{ICC}_{\mathrm{site}}=\frac{\sigma_{\mathrm{site}}^{2}}{\sigma_{\mathrm{site}}^{2}+\sigma_{\mathrm{res}}^{2}}.$$

Values close to 1 indicate that most variation in a weather feature is explained by location, consistent with a site fingerprint. Values close to 0 indicate that little variation is attributable to location. The ICC analysis was applied to raw seasonal weather summaries and to anomaly-transformed weather features. Anomaly features were obtained by removing location-specific baseline conditions:

$$w_{ij}^{*}=w_{ij}-\bar{w}_{i},$$

where $w_{ij}^{*}$ is the anomaly value and $\bar{w}_{i}$ is the location mean for that feature. This comparison was used to determine whether weather predictors in the single-season setting mainly encoded site identity, and whether baseline removal reduced site-level structure.

## Simulated data-generating framework

Simulation analyses were conducted to evaluate study-design conditions that could not be resolved from the single-season empirical dataset alone. The simulations were not intended to estimate absolute field performance, but to examine when weather and microbiome information become identifiable and transferable under strict spatial validation.

Each simulated dataset contained $L$ locations observed across $Y$ years, with $n$ samples collected per location-year. For weather feature $k$, the value for location $l$ in year $y$ was generated as

$$W_{lyk}=B_{lk}+A_{yk}+E_{lyk},$$

where $B_{lk}$ represents the long-term baseline climate of location $l$, $A_{yk}$ represents the seasonal departure shared across locations in year $y$, and $E_{lyk}$ represents residual weather variation. This structure reproduces the empirical observation that raw weather summaries can be strongly location-structured in a single season, while allowing temporal replication to separate baseline site climate from year-specific risk.

Microbiome features were simulated at the sample level to represent realised field state. Features included location-structured variation, weather-responsive components, and residual sample-level heterogeneity. A subset of microbial features was assigned non-zero effects on toxin risk, so that microbiome profiles contained biologically meaningful but imperfect information about the outcome.

Binary mycotoxin risk was generated from a latent linear predictor combining weather-derived seasonal severity, microbiome field state, and location-level heterogeneity:

$$\eta_{i}=\beta_{h}H_{l\left[ i \right]y\left[ i \right]}+\beta_{m}M_{i}+u_{l\left[ i \right]}+\epsilon_{i},$$

where $H_{l\left[ i \right]y\left[ i \right]}$ is the weather-derived severity for the location-year of sample $i$, $M_{i}$ is the microbiome-derived field-state signal, $u_{l\left[ i \right]}$ is a location effect, and $\epsilon_{i}$ is residual variation. The probability of high mycotoxin risk was obtained using the logistic transformation:

$$p_{i}=P\left( Y_{i}=1 \right)=\mathrm{logit}^{-1}\left( \eta_{i} \right)=\frac{1}{1+exp\left( -\eta_{i} \right)}.$$

The binary outcome was then sampled as

$$Y_{i}\sim Bernoulli\left( p_{i} \right).$$

Simulation parameters were chosen to reproduce the qualitative effect-size patterns observed in the empirical analyses, including strong site-structured weather, sample-level microbiome variation, and incomplete but transferable microbiome signal. Simulation results were therefore interpreted as design and identifiability evidence, not as direct validation of future predictive performance.

## Two-stage prediction framework

The simulated analyses were evaluated using a two-stage framework that reflects the timing and biological interpretation of the predictors. Weather variables were treated as early-season information and used to estimate a prior risk score:

$$\eta_{prior,i}=\alpha_{h}+X_{h,i}\beta_{h},$$

where $X_{h,i}$ denotes weather predictors for sample $i$. The corresponding prior risk probability was

$$p_{prior,i}=\mathrm{logit}^{-1}\left( \eta_{prior,i} \right).$$

Microbiome data were treated as in-season observations of realised field state. The weather plus microbiome model updated the weather prior using a microbiome-derived correction fitted from the training data:

$$\eta_{post,i}=\eta_{prior,i}+f_{m}\left( X_{m,i} \right),$$

where $X_{m,i}$ denotes microbiome predictors and $f_{m}\left( X_{m,i} \right)$ is the estimated microbiome update. The final updated probability was

$$p_{post,i}=\mathrm{logit}^{-1}\left( \eta_{post,i} \right).$$

This formulation treats weather as an early-season prior and microbiome composition as a later-season update, rather than assuming that both data streams are available at the same decision point. Microbiome-only models were also fitted to quantify the performance of the in-season biological signal without the weather prior.

## Temporal replication and sampling intensity experiments

All models were evaluated using leave-one-location-out validation. For each fold, all samples from one location were held out as the test set and models were trained on the remaining locations. This procedure directly tested spatial transfer to a previously unseen location.

Two simulation sweeps were performed. In the temporal replication experiment, the number of years per location was varied while holding the number of locations and samples per location-year constant. The simulated values were 1, 2, 3, 5, and 8 years per location. This experiment tested when weather-based risk becomes learnable under spatial holdout.

In the sampling intensity experiment, temporal replication was fixed at three years and the number of samples per location-year was varied from 30 to 100. The number of simulated locations was held fixed at eight. This experiment assessed whether increasing within-year sampling improved discrimination or calibration, and whether performance gains showed diminishing returns.

## Predictive performance metrics

Model discrimination was quantified using the area under the receiver operating characteristic curve. AUC was interpreted as the probability that a randomly selected high-risk sample receives a higher predicted score than a randomly selected low-risk sample:

$$AUC=Pr\left( s_{\text{high}}>s_{\text{low}} \right)$$

where $s_{\text{high}}$ and $s_{\text{low}}$ are the predicted risk scores for high-risk and low-risk samples, respectively. An AUC of 0.5 indicates chance-level discrimination, whereas an AUC of 1 indicates perfect discrimination.

Calibration was evaluated using log loss and Brier score:

$$LogLoss=-\frac{1}{N}\sum_{i=1}^{N} \left[ y_{i}\log\left( p_{i} \right)+\left( 1-y_{i} \right)\log\left( 1-p_{i} \right) \right],$$

$$Brier=\frac{1}{N}\sum_{i=1}^{N} \left( y_{i}-p_{i} \right)^{2}.$$

Lower values of log loss and Brier score indicate better calibrated predictions. For each simulation scenario, results were summarised across Monte Carlo replicates using mean performance and 10-90% quantile intervals.

**Supplementary Figure S3. Extended simulation-based design analysis for integrated weather microbiome prediction.** A, Simulated spatial holdout AUC for microbiome only and weather plus microbiome models across increasing numbers of samples per location per year, with the number of years fixed at three. Points show mean AUC and error bars show 10–90% Monte Carlo intervals. The dotted vertical line indicates the practical knee, where additional sampling gives limited further improvement. B, Corresponding calibration log loss across sample sizes, where lower values indicate better calibration. The extended simulation supports the design interpretation that approximately 40 to 50 samples per location per year provides a practical balance between discrimination, calibration and sampling effort under the simulated assumptions.
